# MS1, a direct target of MS188, regulates the expression of key sporophytic pollen coat protein genes in Arabidopsis

**DOI:** 10.1093/jxb/eraa219

**Published:** 2020-05-06

**Authors:** Jie-Yang Lu, Shuang-Xi Xiong, Wenzhe Yin, Xiao-Dong Teng, Yue Lou, Jun Zhu, Cheng Zhang, Jing-Nan Gu, Zoe A Wilson, Zhong-Nan Yang

**Affiliations:** 1 College of Life Sciences, Shanghai Normal University, Shanghai, China; 2 School of Biosciences, University of Nottingham, Sutton Bonington Campus, Loughborough, Leicestershire, UK; 3 University of Toronto, Canada

**Keywords:** *Arabidopsis thaliana*, MS1, MS188, pollen coat proteins, pollen wall, tapetum

## Abstract

Sporophytic pollen coat proteins (sPCPs) derived from the anther tapetum are deposited into pollen wall cavities and function in pollen–stigma interactions, pollen hydration, and environmental protection. In Arabidopsis, 13 highly abundant proteins have been identified in pollen coat, including seven major glycine-rich proteins GRP14, 16, 17, 18, 19, 20, and GRP–oleosin; two caleosin-related family proteins (AT1G23240 and AT1G23250); three lipase proteins EXL4, EXL5 and EXL6, and ATA27/BGLU20. Here, we show that GRP14, 17, 18, 19, and EXL4 and EXL6 fused with green fluorescent protein (GFP) are translated in the tapetum and then accumulate in the anther locule following tapetum degeneration. The expression of these sPCPs is dependent on two essential tapetum transcription factors, MALE STERILE188 (MS188) and MALE STERILITY 1 (MS1). The majority of sPCP genes are up-regulated within 30 h after MS1 induction and could be restored by *MS1* expression driven by the *MS188* promoter in *ms188*, indicating that MS1 is sufficient to activate their expression; however, additional MS1 downstream factors appear to be required for high-level sPCP expression. Our ChIP, *in vivo* transactivation assay, and EMSA data indicate that MS188 directly activates MS1. Together, these results reveal a regulatory cascade whereby outer pollen wall formation is regulated by MS188 followed by synthesis of sPCPs controlled by MS1.

## Teaser

MS188 and MS1 are essential anther tapetum transcription factors; MS188 directly regulates MS1, which controls the expression of pollen coat protein genes, which are key components of the pollen wall.

## Introduction

In higher plants, pollen is essential for sexual reproduction and for plant survival. Pollen walls help facilitate pollen resilience and are multilayered, with a predominantly gametophyte-derived inner intine layer and a sporophyte-derived outer exine layer, which is further divided into an outer sexine and an inner nexine (Scott and Stead, 1994; Piffanelli and Murphy, 1998; Blackmore *et al.*, 2007; Ariizumi and Toriyama, 2011). The pollen coat, or tryphine, is an extracellular matrix derived from both sporophytes and gametophytes (Doughty *et al.*, 1998), which is deposited onto the pollen grains. During pollen development, microspores are surrounded by a reticulate sexine; the pollen coat later deposits into sexine cavities to complete the pollen wall structure (Piffanelli and Murphy, 1998; Blackmore *et al.*, 2007; Zhou *et al.*, 2015; Wang *et al.*, 2017). The pollen coat has multiple roles, in pollen–stigma interactions, pollen grain hydration, and providing protection from harsh environmental conditions (Preuss *et al.*, 1993; Hülskamp *et al.*, 1995; Pacini and Hesse, 2002). It is mainly composed of saturated acyl groups, proteins, and non-polar esters; the exact composition of the sporopollenin structure has, however, been elusive, partly due to its inert nature. Nevertheless, characterization of pine sporopollenin has recently been possible and this has shown that it is principally composed of aliphatic-polyketide-derived polyvinyl alcohol units and *7-O-p-*coumaroylated C16 aliphatic units, cross-linked through a dioxane moiety (Li *et al.*, 2019). Although many components of the pollen coat are derived from the tapetal cells, other sporophytic anther cell layers and the developing gametophyte are also involved. Recent investigation reveals that very long chain fatty acids (VLCFAs) derived from the endothecium are deposited onto the pollen surface to facilitate pollen hydration (Zhan *et al.*, 2018). Several gametophytic pollen coat proteins (gPCPs) have been reported to be involved in pollen–stigma recognition (Doughty *et al.*, 1993; Takayama, 2000; Nasrallah and Nasrallah, 2014; Wang *et al.*, 2017). SRK (S-locus receptor kinase) is a receptor that allows the stigma to discriminate between genetically related (‘self’) and genetically unrelated (‘non-self’) pollen in the self-incompatibility response of the Brassicaceae. The ligand for SRK [S-locus cysteine-rich protein (SCR)] is a small secreted protein of ~50 amino acids containing eight cysteine residues (Nasrallah and Nasrallah, 2014). SLR1 and SLR2 have been shown to be expressed specifically in the stigmatic papillar cells and to interact with SLR1-binding protein 1 (SLR1-BP1) and SLR1-binding protein 2 (SLR1-BP2); they are both members of the class A PCP family, which includes PCP-A1, an SLG (S locus glycoprotein)-binding protein isolated from *Brassica oleracea* (Doughty *et al.*, 1998; Takayama, 2000). Four Arabidopsis PCP-B-encoding genes, *AtPCP-Bα* (*AT5G61605*), *AtPCP-Bβ* (*AT2G29790*), *AtPCP-Bγ* (*AT2G16535*), and *AtPCP-Bδ* (*AT2G16505*), are expressed gametophytically late in pollen development. Pollen from the triple mutant of these (*pcp-bα/β/γ*) displays a substantially reduced hydration rate on stigmas, delayed pollen tube growth, as well as weakened anchoring to the stigmatic surface (Wang *et al.*, 2017). Various pollen coat components such as proteins and fatty acids are also derived from both the sporophyte and gametophyte, but how the pollen coat genes function and their regulatory relationships are still largely unknown.

Pollen coat proteomics have been investigated in several plants including the Brassicaceae family (Mayfield *et al.*, 2001; Murphy, 2006), *Oryza sativa* (rice) (Dai *et al.*, 2006), *Zea mays* (maize) (Wu *et al.*, 2015), and *Canarium album* (‘olive’) (Rejón *et al.*, 2012). PCPs have been classified into 19 groups (Rejón *et al.*, 2016). Using peptide sequencing in Arabidopsis (*Arabidopsis thaliana*), Mayfield *et al.* (2001) identified 10 PCPs (>10 kDa) based on their high protein abundance in the pollen coat. Among them, five are glycine-rich protein (GRP)–oleosin chimeric proteins (GRP14, GRP16, GRP17, GRP18, and GRP19), which are organized in a cluster with GRP20 on chromosome 5, and two are lipase proteins (EXL4 and EXL6), similarly clustered in a tandem array of six putative lipases on chromosome 1 (EXL1–EXL6) (Mayfield *et al.*, 2001). The remainder include a potential EF-hand Ca^2+^-binding protein (AT1G23240), which is now known as a caleosin-related family protein, and two putative receptor kinases (AT3G21920 and AT4G20670), which are now annotated as cysteine-rich repeat secretory proteins (CRRSP18 and CRRSP41) (Mayfield *et al.*, 2001). These proteins constitute part of the pollen coat family proteins identified in *Brassica napus* (Murphy, 2006), rice (Dai *et al.*, 2006), and olive tree (Rejón *et al.*, 2012). Among these proteins, EXL4 and GRP17 have been shown to be involved in pollen hydration during pollination (Mayfield and Preuss, 2000; Updegraff *et al.*, 2009).

**Fig. 1.**
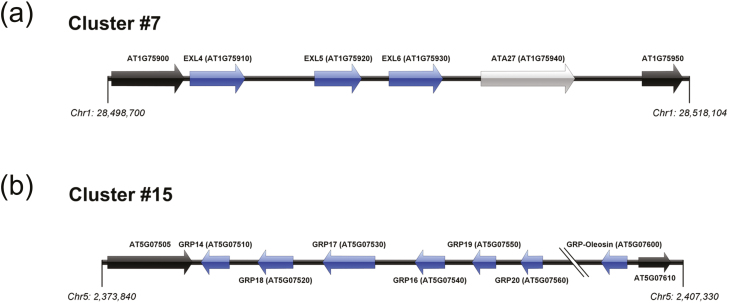
Schematic map of Arabidopsis GRP-related gene regions. Gene clustering of GRP-related genes as classified by Reimegård *et al.* (2017) (a) Extracellular lipases (Cluster #7; loci AT1G75910-940) and (b) glycine-rich proteins (Cluster #15; loci AT5G07510-560 and AT5G07600). Each gene in the clusters is shown as an arrow labelled with the gene name and AGI code. Blue colour indicates the genes that have homology within the cluster, whereas grey indicates lack of homology within the cluster. Black indicates flanking genes that do not respond to MS1 expression, which define the boundary of the cluster. Chromosome coordinates are derived from the TAIR10 Genome browser.

The tapetum is the innermost sporophytic tissue, which is closest to the microsporocytes and is responsible for direct synthesis, as well as release of many pollen coat components after its programmed cell death (PCD). Several transcription factors that are expressed specifically in the tapetum have been functionally characterized (Wilson *et al.*, 2001; Sorensen *et al.*, 2003; Zhang *et al*., 2006, 2007; Zhu *et al.*, 2008), and a genetic pathway [DYT1–TDF1–ABORTED MICROSPORES (AMS)–MALE STERILE 188 (MS188)–MALE STERILITY 1 (MS1)] has been proposed to regulate tapetum development and pollen wall formation (Zhu *et al.*, 2011). In this pathway, the *MS188/MYB80* gene is a key regulator of sexine formation. In *ms188* mutants, the sexine skeleton is absent and there is no pollen coat covering the surface of the pollen wall (Zhang *et al.*, 2007). The *MS1* gene encodes a PHD-finger transcription factor, which functions downstream of *MS188* (Zhang *et al.*, 2007). In *ms1* mutants, abnormal exine bacula are observed, with a lack of vesicles associated with intine formation, suggesting that MS1 is involved in pollen wall and coat formation (Wilson *et al.*, 2001; Ito and Shinozaki, 2002; Vizcay-Barrena and Wilson, 2006; Ito *et al.*, 2007). Reimegård *et al.* (2017) found 17 physically converged gene clusters acting downstream of MS1; Clusters #7 and #15 contain the pollen coat extracellular lipase (EXL4–EXL6) proteins and the GRP–oleosin chimeric proteins, respectively (Fig. 1a, b). Interestingly, these physically clustered and co-regulated genes include not only tandem duplicated genes, but also non-homologous genes. For instance, the *ANTHER27/BETA GLUCOSIDASE 20* (*ATA27/BGLU20*), which locates to Cluster #7, has no homology with any of the adjacent lipases, but shows co-expression with EXL4–EXL6 (Reimegård *et al.*, 2017). *ATA27/BGLU20* has tapetum-specific expression and has been proposed as playing an indispensable role in pollen development (Rubinelli *et al.*, 1998; Dong *et al.*, 2019). ATA27/BGLU20 is predicted to be localized to the endoplasmic reticulum (ER) lumen, which makes this protein less likely to be a sporophytic pollen coat protein (sPCP); however, the co-regulation of this gene with the clustered EXLs may indicate an important role in pollen coat metabolism (Rubinelli *et al.*, 1998). Reimegård *et al.* (2017) speculated that up-regulation of these genes during late tapetum development is due to chromatin conformation changes induced by MS1. However, the relationship between MS1 and these sPCP genes in the tapetum remains unresolved.

In this study, we examined the subcellular localization of six sPCPs (GRP14, GRP17, GRP18, GRP19, EXL4, and EXL6) using translational green fluorescent protein (GFP) fusions and determined that all were initially synthesized in the tapetum, but were later observed to fill the whole locule and surround the microspores. Additionally, we studied the regulatory cascade controlling their synthesis *in vivo*, demonstrating that MS188 directly regulates *MS1*, and MS1 regulates the expression of these sPCP genes, which in turn participate in pollen coat formation. Expression of *MS1* in *ms188* mutants induced rescued expression of these sPCP genes, revealing that MS1 is sufficient to drive the expression of these sPCP genes in the tapetum, although this was not sufficient to rescue normal pollen formation and fertility. Time-course expression analysis using an inducible *MS1-glucocorticoid receptor* (*GR*) line showed that only low levels of sPCP expression could be obtained 30 h post-MS1 induction, suggesting that additional factors, that may be induced by, or at least are downstream of MS1, are needed for late full high-level expression of the sPCPs. This work has revealed the regulatory cascade controlling pollen coat formation and shown that these major sPCPs are synthesized in the tapetum under the control of MS1. It has also indicated that there is temporal regulation of their expression to enable precise control of the biosynthesis of wall materials, and that they are subsequently deposited onto the outer pollen wall regulated by MS188 for functional pollen formation.

## Materials and methods

### Plant materials and growth condition

Arabidopsis accessions Columbia (Col-0) and Landsberg *erecta* (L*er*-0) were used as the wild-type control. Seeds were sown on vermiculite and germinated for 3 d at 4 °C. The plants were grown under 16 h light/8 h dark conditions in a 22 °C growth room. T-DNA insertion mutant lines were obtained from the ABRC and NASC. Transgenic plants were generated via *Agrobacterium tumefaciens*-mediated transformation (Clough and Bent, 1998) and selected on PNS (Plant Nutritional Solution) medium containing 20 mg l^–1^ hygromycin B.

For *ms1* complementation, the 3.476 kb MS1 genomic fragment (993 bp promoter and 2483 bp MS1 gene) was amplified using KOD DNA polymerase (Toyobo) with primer set MS1 com-F/MS1 com-R. The oligonucleotides used for complementation and other assays in this study are listed in Supplementary Table S1 at *JXB* online. Primers were synthesized by Generay. After verification by sequencing (Genomics), the fragment was cloned into the pCAMBIA1300 binary vector (Cambia) and subsequently introduced into the heterozygous +/*ms1* plants, as described previously (Yang *et al.*, 2007). For *ms1* background verification, MWD9-F/MWD9-R primers were used to validate DNA deletion of *MS1* for the transformants, and the genomic-specific primers MS1 JD-F/MS1 JD-R were used to validate the homozygous *ms1* background.

The *pMS1:MS1-GR* line was a kind gift from Dr Peng Qin, Sichuan Agricultural University. A fusion ORF of the MS1 coding sequence (CDS) and GR is driven by a 993 bp upstream sequence of MS1 and followed by a NOS terminator in the inserted T-DNA.

### Expression analysis

The RNA was extracted from Col-0, *ms188*, and *ms1* inflorescences using TRIzol (Life Technologies) following the manufacturer’s instructions. First-strand cDNA was synthesized from 1 μg of total RNA using poly(dT)_12–18_ primer, AMV transcriptase, and accompanying reagents (Takara) for 15 min at 42 °C. Quantitative reverse transcription-PCR (qRT-PCR) was performed using gene-specific primers (see Supplementary Table S1) and SYBR Green Master Mix (Toyobo) on the ABI 7300 platform (Life Technologies), with the program 95 °C for 5 min, 40 cycles of 95 °C for 10 s, and 62 °C for 1 min. Each sample had three replicates and the experiment was repeated three times. The *β-tubulin* gene was used as an internal normalization control. The fold changes in gene expression were calculated according to the ΔCt (cycle threshold) values.

### Dexamethasone induction qRT-PCR

Whole inflorescences with opened flower buds removed were collected for RNA isolation from *ms1-1* and MS1-GR plants [0, 4, 6, 8, 9, 12, 24, and 30 h post-dexamethasone (DEX) treatment] and L*er*-0 wild type. The DEX treatment (spray of 25 μM DEX, 0.01% Silwet L-77) was performed when 0 h inflorescences were sampled. Ten complete inflorescences were collected from a single plant at each time point to represent one single sample. The opened floral buds were removed from the inflorescences. Samples were then preserved in liquid nitrogen in a –80 °C freezer before RNA extraction. Total RNA was extracted from flower buds using the QIAGEN RNeasy Mini Kit (QIAGEN GmbH, Hamburg, Germany) in accordance with the manufacturer’s instructions. Three biological replicates and two technical replicates were used to set up the qRT-PCRs.

### ChIP

The ChIP procedure was performed on flower buds of *pMS188:MS188-GFP* and *pMS188:4×MYC-MS188* transgenic lines as described by Lou *et al.* (2014) with minor modifications. The chromatin solution was incubated overnight with the GFP and MYC antibody (Millipore) at 4 °C. A total of 0.8–1.0 g of inflorescence tissue of the wild-type plant was collected and cross-linked in formaldehyde buffer. After isolating nuclei and shearing the chromatin by ultrasonication, the majority of the DNA fragments were between 200 bp and 800 bp. After pre-immune serum treatment with sheared salmon sperm DNA/protein A agarose mix (Millipore, USA) for 1 h, the supernatants were incubated with polyclonal antibody against AMS (GL Biochem, China) at 4 °C overnight with 1:100 dilution. A 40 µl aliquot of magnetic beads–protein G (Invitrogen) was added to precipitate the antibody–protein/DNA complexes. The DNA fragments were eluted after reverse cross-linking at 100 °C for 10 min. The remaining steps for purification of DNA were carried out according to the manufacturer’s instructions. Quantitative PCR (qPCR) was performed on an ABI PRISM 7300 detection system (Applied Biosystems, USA) with SYBR Green I master mix (TOYOBO, Japan). All PCR experiments were performed under the following conditions: 95 °C for 5 min, 40 cycles of 95 °C for 10 s, and 60 °C for 1 min; the ΔCt values (Ct of each sample–Ct of the no antibody control) were calculated and 2^–ΔCt^ was taken as the fold enrichment. Primers for ChIP-qPCR analysis are listed in Supplementary Table S1.

### EMSAs

To obtain the maltose-binding protein (MBP)–MS188 protein for the EMSA experiments, the full-length fragment of the *MS188* gene was amplified using the primer pair MS188PMAL-F/R cloned into the pMAL-c5× vector (GE Healthcare). The fusion protein was expressed and purified according to the manufacturer’s instructions. The DNA fragment containing the MYB-binding site in the *MS1* promoter was amplified and a biotin-labelled and competitor probe, named probe *MS1-1* and *MS1-2*, were produced. EMSA was performed with a LightShift Chemiluminescent EMSA Kit (Thermo Scientific) according to the manufacturer’s instructions. The relevant primer sequences are listed in Supplementary Table S1.

### Dual-LUC transient transactivation assays in tobacco (*Nicotiana benthamiana*) leaves

For the dual-luciferase (Dual-LUC) assay, the pGreenII 0800 LUC plasmid (Hellens *et al.*, 2005) was modified as the effector vector. A Gateway R1R2 cassette and a 66 bp mini 35S promoter were PCR amplified and sequentially inserted into the multicloning site (MCS) upstream of the firefly luciferase gene in the effector vector, to make it compatible for the Gateway cloning technique and capable to test promoter sequences lacking a TATA-box or other essential elements of transcription. The Probe-1^MS1^ sequence was first TA TOPO (Thermo Fisher Scientific) cloned into pCR8 and LR cloned into the effector vector. The *MS188* coding sequence was Gateway cloned into the pUB-DEST vector (Grefen *et al.*, 2010). All vectors were co-infiltrated into tobacco leaves with p19 of *Tomato bushy stunt virus* to enhance the transient expression. The infiltrated leaf tissues were collected 3 d after transfection and assayed for the LUC and REN levels using the Promega Dual-Glo® Luciferase Assay Kit (E2920) and BioTek Synergy LX Microplate Reader in accordance with the manufacturer’s instructions.

### Rescue experiment

A 1439 bp *MS188* promoter fragment was amplified and cloned into the modified GFPpCAMBIA1300 vector. Then a 2483 bp genomic sequence of *MS1* was amplified and inserted into the above plasmids. After confirmation, the *A. tumefaciens* GV3101 containing the *pMS188:MS1* plasmid was transformed into the heterozygous +/*ms188* plants. Transgenic plants were generated via *A. tumefaciens*-mediated transformation (Clough and Bent, 1998) and selected on PNS medium containing 20 mg l^–1^ hygromycin B. Primers sequences are listed in Supplementary Table S1.

### Accession numbers

Sequence data were archived in TAIR (www.arabidopsis.org) and the National Centre for Biotechnology Information with the following accession numbers: *MS1* (AT5G22260), *MS188*/*MYB80*/*MYB103* (AT5G56110), *MYB99* (AT5G62320), *EXL4* (AT1G75910), *EXL5* (AT1G75920), *EXL6* (AT1G75930), *ATA27/ BGLU20* (AT1G75940), *Caleosin* (AT1G23240), *CRRSP18* (AT3G21920), *CRRSP41* (AT4G20670), *GRP14* (AT5G07510), *GRP16* (AT5G07540), *GRP17* (AT5G07530), *GRP18* (AT5G07520), *GRP19* (AT5G07550), *GRP20* (AT5G07560), and *GRP-Oleosin* (AT5G07600).

## Results

### Multiple abundant pollen coat proteins are synthesized in the tapetum

**Fig. 2.**
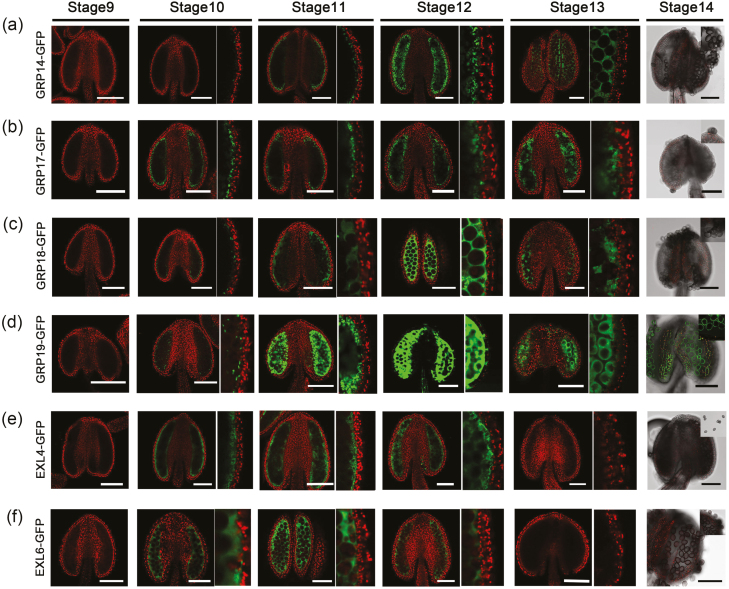
Localizations of sPCPs in anther. Fluorescence confocal images of the GRP14–GFP (a), GRP17–GFP (b), GRP18–GFP (c), GRP19–GFP (d), EXL4–GFP (e), and EXL6–GFP fusion proteins (f). The green channel showed the GFP expression (530 nm) and the red channel showed chlorophyll autofluorescence (>560 nm). Anther stages are based upon Sanders *et al.* (1999). Scale bars=100 μm.

In a previous study, highly abundant PCPs in Arabidopsis were identified from analysis of the pollen surface by SDS–PAGE (Mayfield *et al.*, 2001). Studies also showed that GRP17 fused with GFP was located in the tapetum (Mayfield *et al.*, 2001; Suzuki *et al.*, 2013), and that a GRP19–GFP protein fusion derived from the tapetum is ultimately deposited onto the pollen grain surface (Lévesque-Lemay *et al.*, 2016), which indicates that GRP17 and GRP19 are both sporophytic in origin. However, tissue origins and subcellular locations of other PCPs are still uncharacterized. To confirm the expression patterns of these PCPs, we generated *pPCPs:PCPs-GFP* constructs, named PCP–GFPs, which were driven by their respective native promoters, and transformed into wild-type plants. GFP signals of all transgenic lines showed similar expression patterns, with initial detection in tapetal cells at the start of tapetum degeneration (stage 10), followed by high accumulation in the locule at the time of tapetum PCD (stages 11–12) (Fig. 2). However, subtle differences in patterns of expression were seen between the various PCP–GFP fusions, with minimal expression of GRP14 and GRP18 observed at stage 10, compared with GRP17, EXL4, EXL6, and GRP19. Signals of EXL4–GFP and EXL6–GFP were undetectable at the pollen tricellular stage (stage 13), although other PCPs still showed signal in the locule at this stage (Fig. 2e, f). Signals were also undetectable on pollen grain surfaces after anther dehiscence, except for GRP19–GFP (Fig. 2d). In addition, no GFP signals were present inside microspores during pollen development (Fig. 2). These data indicate that these PCPs are derived from the tapetum, hence they are classified as sPCPs, and that there is differential expression and potential deposition into the anther locule.

### *MS1* acts upstream of multiple pollen coat protein genes

**Fig. 3.**
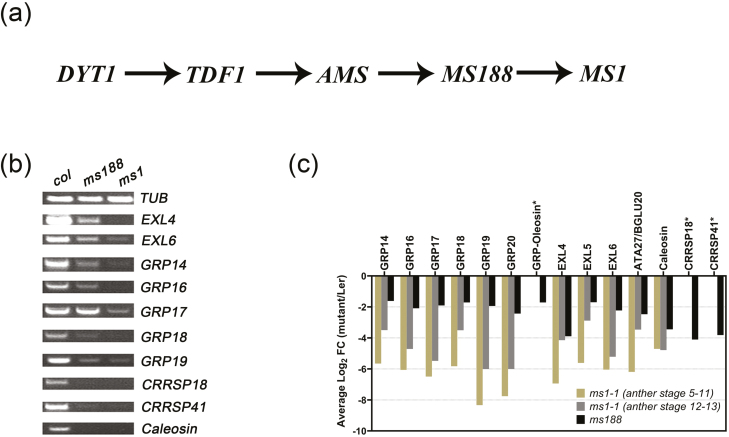
sPCP genes are downstream of *MS1.* (a) The genetic pathway of key transcription factors for tapetum development and function. (b) Expression analysis of 10 sPCP genes was determined in inflorescences of wild-type (Col-0), *ms188*, and *ms1* plants by RT–PCR analysis using 28 cycles; the *β-tubulin* gene was used as an internal normalization control. (c) Log_2_-fold change values of the selected sPCPs. Values are extracted from previously published microarray data from the *ms188* (Li *et al.*, 2017) and *ms1* mutants (Yang *et al.*, 2007), and converted to the average log_2_ ratio of the mutant expression compared with that of the wild type. An asterisk denotes the genes that do not have an expression value in the MS1 microarray due to lack of specific detection probes in the Arabidopsis Genome ATH1 Array (Pearce *et al.*, 2015). All genes shown have expression levels in the mutant significantly different from the wild type (the calculated log_2_ ratio has *q*<0.01)

A number of tapetally expressed transcription factors (DYT1, TDF1, AMS, MS188, and MS1) have been identified as essential for tapetum development and pollen formation (Wilson *et al.*, 2001; Sorensen *et al.*, 2003; Zhang *et al*., 2006, 2007; Zhu *et al*., 2008, 2011, Fig. 3a). MS1 is the final regulator identified in this pathway; in *ms1* mutants, a reduction of GRP expression occurs and pollen coat formation is not observed (Yang *et al.*, 2007), suggesting that the sPCP genes act downstream of *MS1*. To confirm this, RT–PCR assays were performed on flower buds from Arabidopsis wild type and *ms188* and *ms1* mutants. Results showed that expression of most sPCP genes was decreased in both *ms1* and *ms188*, while the expression of *CRRSP18*, *CRRSP41*, and *Caleosin* was barely detectable in both *ms188* and *ms1* (Fig. 3b). A re-analysis of the *ms1* and *ms188* microarray data further confirmed down-regulation of the sPCPs in these two mutants (Fig. 3c). It is worth noting that these sPCPs showed less down-regulation in the *ms188* mutant compared with *ms1*, based on the microarray data (Fig. 3c), suggesting that MS1 has a stronger regulatory impact on the sPCP genes compared with MS188. There were also differences seen in the stages and extent of down-regulation of the expression of the different GRPs in *ms1*, suggesting that they are also under differential regulation.

### Induction of MS1 restores the expression of sPCP genes

**Fig. 4.**
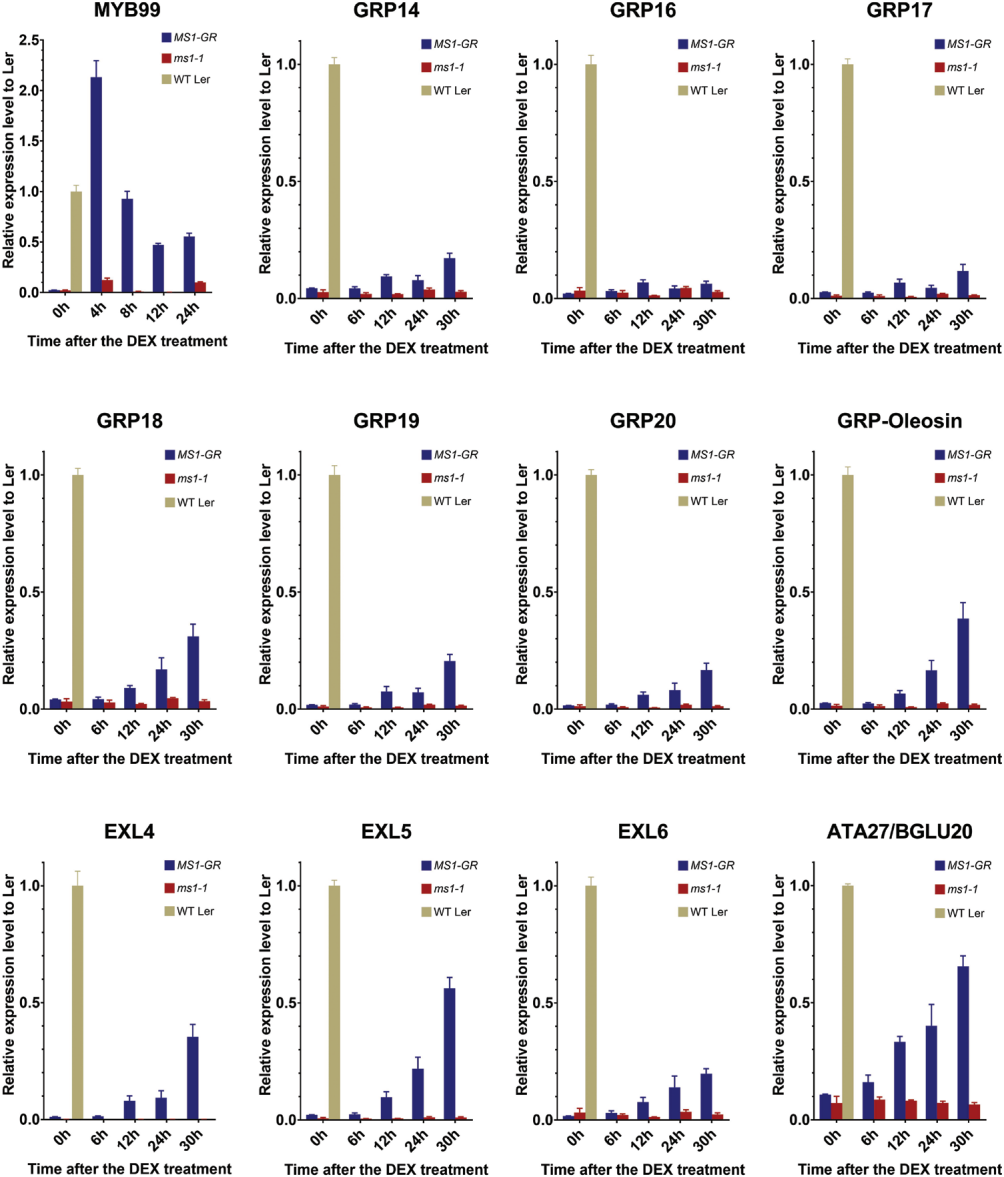
MS1-DEX-induced gene expression of the *sPCP* genes and *MYB99.* Time-course qRT-PCR detection of the rapid induction of gene expression of the direct MS1 target *MYB99* at 4, 8, 12, and 24 h, and *sPCP* gene expression at 6, 12, 24, and 30 h post-DEX treatment in the flower buds from the transgenic line *pMS1:MS1-GR* (*ms1-1 Ler*-0 background) and the *ms1-1* (L*er*-0) mutant, respectively. Expression at 0 h is from flower buds collected just before DEX treatment. The relative expression levels of each target gene are normalized to its wild-type L*er*-0 flower expression and are presented as means ±SEM.

Reimegård *et al.* (2017) previously reported that the GRP–oleosin chimeric genes physically clustered on chromosome 5 (cluster #15, Fig. 1b) are up-regulated within 48 h after MS1 induction in plants expressing MS1–GR fusion protein. To determine in more detail the dynamics of the transcriptional responses of sPCPs to MS1, we carried out a time-course qRT-PCR analysis of the expression of sPCPs after DEX induction of MS1 using a *MS1-GR* line (*ms1-1*/*pMS1:MS1-GR*). *MYB99* was used as a positive control for MS1 induction since it has been previously reported as a direct regulatory target of MS1 (Alves-Ferreira *et al.*, 2007; Ito *et al.*, 2007). Induction of MYB99 was very rapid and high level, reaching more than twice the wild-type expression levels 4 h post-DEX treatment (Fig. 4). However, the induction of EXL4 and EXL6, the GRPs, except for GRP16 which showed minimal changes, and ATA27, was observed much later, 12–30 h post-DEX treatment, with a relatively low level of induction (15–60%) compared with the wild type (Fig. 4). ATA27 showed a stronger, earlier induction starting at 6 h, whilst caleosin and CRRSPs did not respond to MS1-DEX induction within 30 h (Supplementary Fig. S1). This indicates that although both MYB99 and these sPCPs act downstream of MS1, activation of their expression is differentially regulated. The delay in induction of expression of the sPCPs suggests that additional transcription factors, possibly induced by MS1, for example MYB99, may be required to facilitate the full expression of these sPCP genes. This may serve as a mechanism to introduce temporal regulation of the synthesis of components of the pollen wall and to orchestrate the complex deposition and structure of the pollen wall.

### MS188 is a direct upstream regulator of *MS1*

**Fig. 5.**
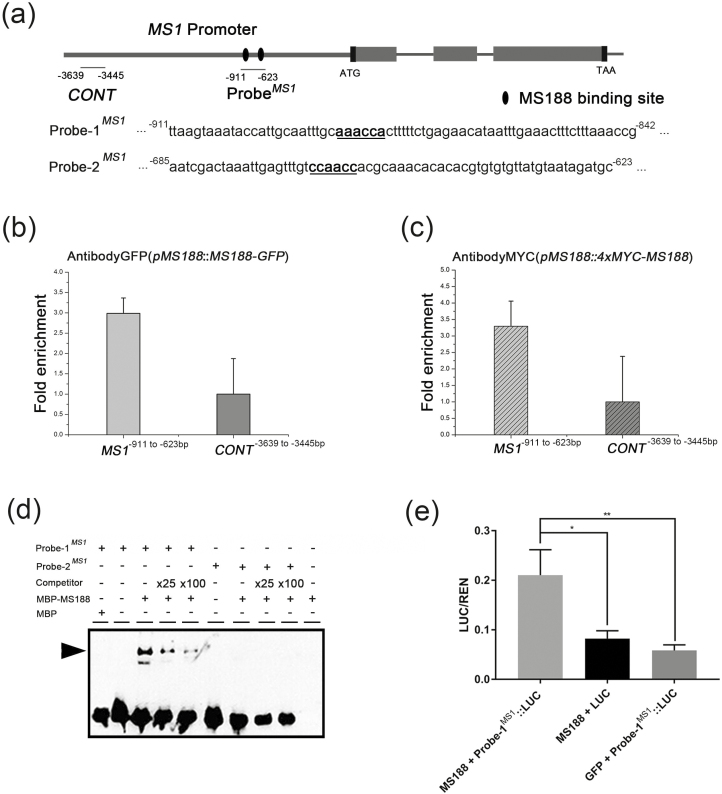
MS188 directly regulates *MS1 in vivo* and *in vitro.* (a) Potential MYB-binding sites in the promoter and genome regions of the *MS1* gene. Black ellipses indicate the potential MS188-binding sites. Probe^*MS1*^ contains these binding sites. (b, c) The enrichment of the *MS1* promoter was confirmed by ChIP-qPCR with primer sets [MS1^−911 to −623 bp^, CONT^MS1(−3639 to −3445 bp)^] using the *pMS188:MS188-GFP* (b) and the *pMS188:4×MYC-MS188* (c) samples. The fold enrichment was calculated from three independent replicates. Error bars represent the SD (*n*=3). (d) Probe^*MS1*^ was divided into two segments containing the MYB-binding site named Probe-1^*MS1*^ (MS1^−911 to −842 bp^) and Probe-2^*MS1*^ (MS1^−685 to −623 bp^). MBP–MS188 protein was mixed with a biotin-labelled probe, a 25-fold and 100-fold unlabelled competitor probe for EMSA assay. The arrowhead indicates a band shift. (e) Dual-LUC assay measuring the MS188 transactivation on the Probe-1^*MS1*^-driven LUC reporter. The MS188 effector (*AtUbi10:MS188*) paired with empty LUC reporter and free GFP (*AtUbi10:GFP*) paired with the Probe-1^*MS1*^-driven LUC reporter were co-transfected to serve as controls. One-way ANOVA and pairwise Dunnett test were used to test the statistical difference between groups (*P<0.05, **P <0.01). Error bars stand for Probe-1 SD.

*MS1* is thought to act downstream of *MS188* in the tapetum genetic pathway (Zhang *et al.*, 2007; Zhu *et al.*, 2011); however, the nature of this relationship is unknown. A ChIP assay was therefore conducted to investigate whether MS188 directly regulates *MS1*. *MS188* codes for a MYB transcription factor; AACC forms the core of the MYB1AT *cis*-element bound by MYB2 with a consensus sequence of CTAACCA (Abe *et al.*, 1997). It was reported to recognize CCAACC/AAACCA/CTAACCT as *cis*-elements for MS188 and also the AACC consensus sequence (Phan *et al.*, 2011; Xiong *et al.*, 2016). The 993 bp upstream region of MS1 driving *MS1-GFP* was able to complement the *ms1* mutant phenotype (Supplementary Fig. S2), suggesting that this region contains motifs for upstream regulator recognition. This region contains two putative MYB-binding sites AAACCA (–886 to –881) and CCAACC (–664 to –659) (Fig. 5a). In our previous study, we constructed *pMS188:MS188-GFP* and *pMS188:4xMYC-MS188* transgenic complementary lines (Xiong *et al.*, 2016). To investigate the interaction of the MS188 protein with the *MS1* promoter *in vivo*, we performed a ChIP assay using buds from transgenic plants. ChIP-qPCR results indicated that an upstream region of MS1 (base pairs −911 to −623), containing the predicted MS188-binding sites (CCAACC and AAACCA), was enriched (Fig. 5b, c). On the other hand, when a distal upstream region (base pairs −3639 to −3445, CONT) of *MS1* was ChIP-qPCR amplified, no obvious enrichment was observed (Fig. 5b, c). A recombinant MBP–MS188 protein (Supplementary Fig. S3) and two probes containing the MS188-binding motif were used for EMSA (Fig. 5a). The results showed that MS188 binds to Probe-1^*MS1*^ (AAACCA) but not Probe-2^*MS1*^ (CCAACC) (Fig. 5d). When the unlabelled probe was added, the excess competitor reduced the abundance of shifted bands in a concentration-dependent manner, further confirming the binding specificity (Fig. 5d). To further detect whether MS188 can directly activate *MS1 in vivo*, we carried out a quantitative Dual-LUC transactivation assay, in which an MS188 effector vector, a dual-reporter vector possessing both Probe-1^*MS1*^-driven firefly luciferase (LUC) and the 35S-promoter driven Renilla luciferase (REN), and a helper vector expressing the p19 silencing suppressor, were co-transformed into *N. benthamiana* leaves. The MS188 transactivation level of Probe-1^*MS1*^ was measured by normalizing the signal of the experimental LUC reporter to that of the internal control REN. By using a constitutively expressed REN reporter as an internal reference reporter, the experimental variability caused by differences in cell transformation efficiency and viability was minimized (Lasierra and Prat, 2018). Our Dual-LUC assay showed that the effector–reporter combination of MS188–Probe-1^*MS1*^ exhibited a statistically significant higher transactivation level compared with negative controls (Fig. 5e). These results together indicate that MS188 directly binds to the specific site of the *MS1* promoter and directly activates *MS1* expression in the tapetum.

### *MS1* driven by the *MS188* promoter restores the expression of multiple pollen coat protein genes in *ms188* mutants

**Fig. 6.**
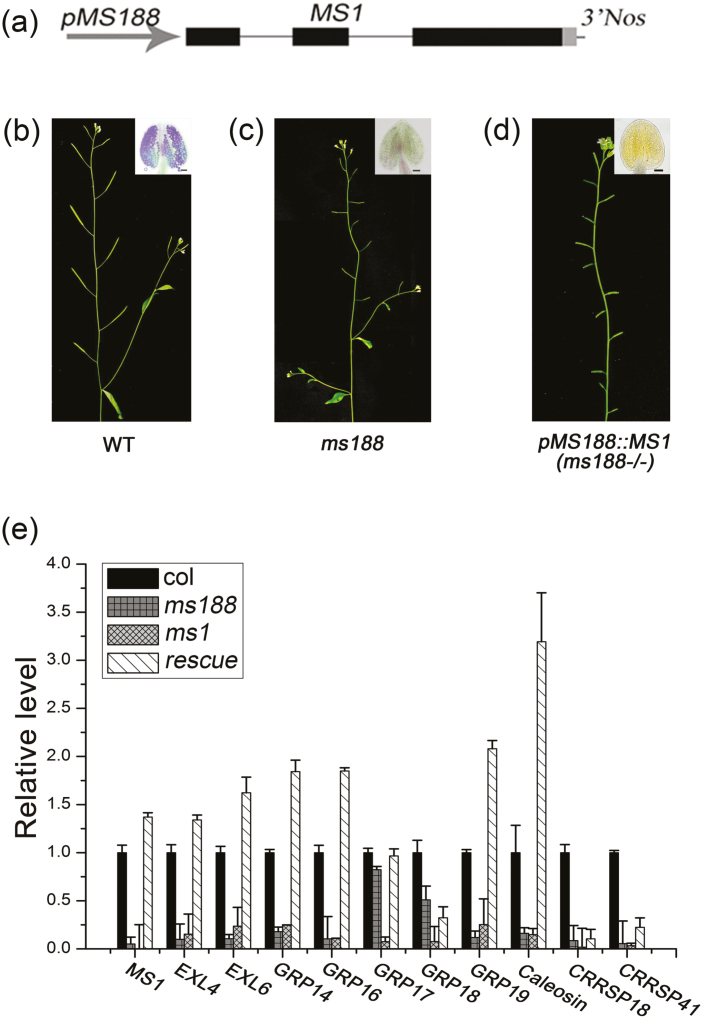
Expression of most sPCP genes can be fully recovered in *pMS188:MS1* transgenic plants in the *ms188* homozygous background. (a) The *pMS188:MS1* construct used for genetic complementation assay. (b–d) A wild-type plant with normal fertility, an *ms188* plant, and a totally male-sterile *pMS188:MS1* rescue plant, respectively. Inserts show anthers stained for viable pollen from the lines. Scale bar= 20 µm. (e) The *sPCP* gene expression analysis by qRT-PCR in Col-0, *ms188*, *ms1*, and the *pMS188:MS1* rescue plant. Error bars show the SD (*n*=3).

The expression of several sPCPs was absent or decreased in both the *ms188* and *ms1* mutants, and induction of MS1 increased expression of many of the sPCPs (Fig. 4); however, rescue was not to wild-type levels. Whether MS188, or other factors, were necessary for expression of these sPCPs was unknown, therefore we expressed MS1 in the *ms188* mutant background to examine whether MS1 is sufficient for rescue of sPCP gene expression in the tapetum. We generated a *pMS188:MS1* construct and transformed it into heterozygous +/*ms188* plants (Fig. 6a). Similar to the *ms188* plants (Fig. 6b, c), all transgenic lines with a homozygous *ms188* background exhibited male sterility (Fig. 6d). To characterize the expression of the sPCP genes, we performed qRT-PCR using buds from wild-type, *ms1*, and *pMS188*:*MS1* transgenic plants. The expression of *MS1* was restored in *pMS188*:*MS1* transgenic plants (Fig. 6e). Fertility of these transgenic lines was not rescued by the *pMS188*:*MS1* transgene, and no mature pollen formation was seen (Fig. 6d), indicating that MS188 and potentially additional downstream factors in the MS188 network are required for functional pollen development. Nevertheless, qRT-PCR analysis showed that most of the expression of the sPCP genes was completely restored in *pMS188*:*MS1* transgenic plants (Fig. 6e). Therefore, these results show that *MS1* is a major regulator for the activation of most of the sPCP genes; however, the delay in induction seen with the DEX-inducible qRT-PCR data implies that there may be additional regulators that MS1 controls in the native state, which are required to enable high levels of sPCP gene expression to be generated. These data suggest that the additional factors implicated as required to control high-level downstream sPCP expression are regulated by MS1 rather than the direct MS188 network.

## Discussion

### PCPs and their functions

After pollen lands on the stigma, it adheres, hydrates, and germinates to produce a pollen tube inside the stigma; the pollen coat plays a critical role in facilitating this process (Edlund *et al.*, 2004). The pollen coat contains various components, which include PCPs synthesized from the tapetum alongside gametophytically derived proteins, including SCR, PCP-A1, SLR-BP, and PCP-Bs (Doughty *et al.*, 1998; Takayama, 2000; Nasrallah and Nasrallah, 2014). In Arabidopsis, 10 PCPs including a potential caleosin-related family protein, two putative receptor kinases (CRRSP18 and CRRSP41), two lipase proteins, and five GRPs have been identified as highly abundant proteins in the pollen coat (Mayfield *et al.*, 2001). They have also been identified in the pollen coat of other plant species (Huang *et al.*, 2013). Two of these proteins (EXL4 and GRP17) have been reported to be involved in pollen hydration (Mayfield and Preuss, 2000; Updegraff *et al.*, 2009). These sporophyte-derived PCPs may play roles in the early events of pollen–stigma recognition including pollen adhesion and hydration. In addition, endothecium-derived VLCFAs, which cover the surface of the exine, also facilitate pollen hydration (Zhan *et al.*, 2018).

In recent years, a number of gametophyte-derived PCPs, including S-locus cysteine-rich protein (SCR), SLR1-BP1/2, PCP-A1(PCP7), and PCP-Bs, have also been identified. Arabidopsis pollen coat cysteine-rich proteins (CRPs), the PCP-Bs, have been reported to be involved in pollen hydration (Wang *et al.*, 2017). In the Brassicaceae family, SCR has been shown to interact with S-locus Receptor Kinase (SRK) (Nasrallah *et al.*, 2014). Pollen coat proteins SLR1-BP1 and SLR1-BP2 have been shown to bind S locus-related glycoprotein1 (SLR1) in *Brassica campestris* (Takayama *et al.*, 2000), and PCP-A1 acts as a ligand interacting with S-locus glycoprotein (SLG) (Doughty *et al.*, 1993).

During pollen wall development, the microsporocytes and tapetum work in tandem to facilitate final pollen wall generation. The tapetum goes through a highly active stage of pollen wall material synthesis and then undergoes PCD. On the other hand, *in situ* hybridization data have shown that at stage 10 the PCP-A1 and PCP-B gPCPs are produced by tricellular microspores (Doughty *et al.*, 1998; Wang *et al.*, 2017). These data indicate that the multi-type PCPs in tryphine are produced in spatio-temporal regulation during late pollen development. Pollen germination is a late event in pollen–stigma recognition; therefore, these gametophyte-derived PCPs are likely to play important roles in pollen germination. However, tapetum transcription factors, such as MS1, which are critical for the formation of viable pollen, may also play an important role in the correct deposition of the pollen coat to help determine the subsequent hydration and germination of pollen.

### The expression and localization of the major sporophytic pollen coat proteins

Thirteen sPCPs have been identified in Arabidopsis; however, the detailed expression pattern of only GRP17 has been determined; GRP17–GFP was detected initially in the tapetum and later in anther locules (Suzuki *et al.*, 2013). In this study, we confirm this expression pattern for GRP17 and show that the other sPCPs exhibit similar expression patterns, in which the major fluorescence signals were initially detected in tapetum cells at stage 10, and gradually reach a peak level of signal in the locule surrounding the microspores at the later stages 11–12 (Fig. 2). These results strongly support the hypothesis that these sPCPs are initially synthesized in the tapetum and subsequently exported, or released into the anther locule following tapetum degeneration. However, there were differences observed between the expression patterns of the various sPCPs, suggesting an actively controlled process. We observed EXL4–GFP and EXL6–GFP signals earlier, and these were sustained for a shorter period compared with others (Fig. 2e, f). EXL4 and EXL6 contain the predicted family II lipase domain, which performs acyl transfer reactions in extracellular environments (Upton and Buckley, 1995). These two lipases may function in the long chain fatty acid modification process before the precursors of pollen coat are deposited onto the pollen surface. Fusion proteins GRP14–GFP, GRP17–GFP, GRP18–GFP, and GRP19–GFP were localized in locules until stage 13. However, signals were not observed on the surface of pollen grains, except for GRP19–GFP (Fig. 2a–d). It is reported that GRP17–GFP is cleaved between the GRP17 and GFP at stage 12; therefore, fluorescent signals on the pollen surface at stage 14 are derived from the cleaved fusion protein fragment possessing intact GFP (Suzuki *et al.*, 2013). Similar cleavage of GRP19–GFP has been previously reported, but the pollen surface GFP signals are much stronger than GRP17–GFP (Lévesque-Lemay *et al.*, 2016). These results suggest that cleavage of fusion proteins may also be occurring in our transgenic lines. Hence, failure to detect a pollen surface GFP signal may have resulted from low sensitivity of the confocal microscope, or due to the fact that specific cleavage of the GFP protein had occurred.

Previous research work showed that CYP703A2 catalyses the monohydroxylation of medium chain saturated fatty acids essential for sporopollenin synthesis (Morant *et al.*, 2007). CYP703A2–GFP and some other sporopollenin synthesis-like proteins, ACOS5–GFP, TKPR1–GFP, TKPR2–GFP, and PKSA–GFP, were initially detected in the tapetum at stage 7 and then were released into the locule where their highest accumulation was observed (Xiong *et al.*, 2016; Wang *et al.*, 2018). Thus, sporopollenin synthesis enzymes exhibit a similar localization pattern to that of the sPCPs; however, expression was much earlier. CYP703A2–GFP was hardly detected at stage 10 (Xiong *et al.*, 2016), while we found that most PCP–GFPs accumulated at stages 11–12 (Fig. 2). It is generally thought that tapetum degeneration initiates at stage 10 and is finally complete by stage 12 (Sanders *et al.*, 1999). Therefore, CYP703A2, and potentially other sporopollenin synthesis enzymes, are likely to be secreted earlier during stage 7 into the locule by the tapetum for sporopollenin synthesis and sexine formation. In contrast, sPCPs may be transferred from the tapetum into the locule later through a secretory pathway and/or during subsequent tapetum degeneration.

### MS1 mediates the expression of a set of sporophytic pollen coat protein genes

The *ms1* mutant was the first reported male-sterile line in Arabidopsis (van der Veen and Wirtz, 1968). MS1 is critical for pollen development during late anther development (Wilson *et al.*, 2001). In *ms1*, the exine is aberrant and the pollen coat is completely absent (Vizcay-Barrena and Wilson, 2006). Pollen coat is derived from the tapetum at least partly under the control of sporophytic genes (Piffanelli *et al.*, 1998); however, the regulatory mechanism for pollen coat formation remains unknown. A recent study showed that after *pAMS:AMSGR-YFP* plants were treated with DEX, *GRP18* and *EXL6* were induced after 48 h, while *MS188* was induced immediately, suggesting that MS188 may be a direct target of AMS, whilst sPCP expression occurs much later, possibly requiring additional factors for their expression (Ferguson *et al.*, 2017). This agrees with our results that MS1 mediates the expression of a set of sPCPs, and with previous investigations indicating that most sPCP genes are downstream of MS1 (Ito *et al.*, 2007; Yang *et al.*, 2007). Previous investigations demonstrated that AMS can bind to the promoters of *EXL4*, *EXL6*, *GRP14*, *GRP18*, and *GRP19* (Xu *et al.*, 2014). In *ms188*, the expression of these genes was significantly reduced (Fig. 3). However, AMS expression was not affected (Zhu *et al.*, 2008). Therefore, AMS is not the principal regulator of these sPCP genes, although it may bind to the promoters of these genes and be involved in moderating their expression. Our data show that when MS1 was driven by the MS188 promoter, the expression of many of the sPCPs was completely restored, although viable pollen was not formed. This, and the delay in induction (Fig. 4), implies that there are other factors that may be involved in the sPCP regulation pathway, which are not directly induced by MS188. However, the identity of these factors and whether they function dependently or independently of MS1 is currently unknown.

### The tapetum regulatory cascade and pollen formation

**Fig. 7.**
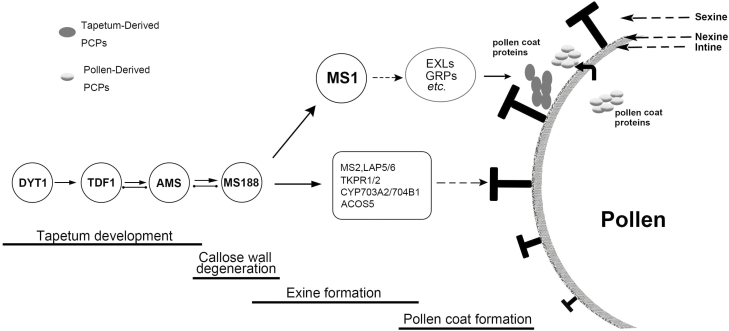
Proposed model for the tapetum regulatory cascade required for tapetum development and pollen formation. The complex regulatory network subtly controls a series of developmental events in the Arabidopsis anther. DYT1, TDF1, and AMS are required for the early tapetum development. AMS also initiates sexine formation via activating *MS188*. Later, MS188 interacts with AMS to regulate sporopollenin precursor synthesis genes and *MS1* for sporopollenin precursor synthesis. After initiation of the exine skeleton, MS1 and/or other factors regulate/co-regulate sPCP genes for pollen coat synthesis in the tapetum. The black solid line arrows indicate positive transcriptional regulation. Dashed line arrows show the function of these proteins involved in pollen wall formation. The black solid line with two dots indicates protein interactions.

During anther development, the tapetum acts in multiple roles in pollen formation, providing nutrition for microspore development, secreting hydrolases for tetrad callose wall dissolution, and supplying structural components such as sporopollenin and pollen coat for pollen walls (Piffanelli *et al.*, 1998; Li and Li, 2012). The genetic pathway DYT1–TDF1–AMS–MS188–MS1 is important for tapetum development (Zhu *et al.*, 2011), in which *TDF1* is a target of DYT1 (Gu *et al.*, 2014), *AMS* is a target of TDF1 (Lou *et al.*, 2018), and *MS188* is a target of AMS (Lou *et al.*, 2014), although it is clear that the complexities of these relationships are significant and are key to controlling the correct deposition of pollen wall materials (Ferguson *et al.*, 2017). Here, we identified that *MS1* is a direct target of MS188 (Fig. 5). Therefore, this pathway represents a regulatory cascade that serves to temporally control multiple tapetum functions (Fig. 7). In this pathway, AMS has a significant role as a regulator to initiate sexine and nexine formation (Lou *et al.*, 2014; Xu *et al.*, 2014), whilst also regulating *ABCG26* for sporopollenin transport (Xu *et al.*, 2010). In addition, AMS directly regulates *MGT5* to provide the Mg^2+^ for microspore development (Xu *et al.*, 2015). Sexine is the outer pollen wall where pollen coats (including PCPs) are deposited. MS188 directly regulates *CYP703A2* and other sporopollenin biosynthesis genes (SSGs) for sporopollenin synthesis and sexine formation (Xiong *et al.*, 2016; Wang *et al.*, 2018).

MS1 as the final transcription factor in this pathway is involved in the control of several events such as tapetum PCD and tryphine synthesis in late pollen maturation (Vizcay-Barrena *et al.*, 2006; Yang *et al.*, 2007). Our work suggests that MS188 directly regulates MS1, which subsequently mediates the expression of sporophytic PCP genes; however, their regulation is clearly under temporal control through the requirement for additional downstream factors (Fig. 7). Therefore, sporophyte-derived PCPs are synthesized after the sexine is formed. This regulatory pathway therefore represents a staged developmental process whereby outer pollen wall formation is regulated by MS188 and then followed by sporophytic PCP synthesis mediated by MS1. Previous studies have shown that MS1–GFP signal was detected at the tetrad/microspore release stage (Yang *et al.*, 2007). Our data confirm this result (Supplementary Fig. S2), and the observed delay of the expression of these PCPs and lag of induction after DEX induction of MS1 suggests that additional factors are required to initiate high-level PCP expression (Fig. 7). Together, these results reveal a regulatory cascade whereby outer pollen wall formation is regulated by MS188, followed by sPCP synthesis controlled by MS1, but mediated by other factors downstream of MS1. This may therefore serve as a mechanism to temporally regulate the deposition of pollen wall components to generate the complex final pollen wall structure.

## Supplementary data

Supplementary data are available at *JXB* online.

Fig. S1. sPCP genes that showed no significant up-regulation 30 h post-DEX induction in the MS1-GR line.

Fig. S2. MS1 expression in *pMS1:MS1-GFP* complementation plants.

Fig. S3. Expression and purification of MS188 proteins.

Table S1. Primers used in this study.

eraa219_suppl_Supplementary-Data-Files

## Abbreviations

ACOS5: ACYL-COA SYNTHETASE
AMS: ABORTED MICROSPORES
ATA27/BGLU20: ANTHER 27/BETA GLUCOSIDASE 20
Col-0: Columbia
CRRSP: CYSTEINE-RICH REPEAT
EXL4/5/6: EXTRACELLULAR LIPASE
GFP: green fluorescent protein
gPCP: gametophytic pollen coat protein
GR: glucocorticoid receptor
GRP14/16/17/18/19/20: GLYCINE-RICH PROTEIN 14/16/17/18/19/20
L*er*-0: Landsberg *erecta*
MGT5: MAGNESIUM TRANSPORT 5
MS1: MALE STERILITY 1
MS188: MALE STERILE188
PCD: programmed cell death
PCP: pollen coat protein
PKSA: POLYKETIDE SYNTHASE A
SCR: S-LOCUS CYSTEINE-RICH PROTEIN
SLG: S LOCUS GLYCOPROTEIN
SLR1: S-LOCUS RELATED PROTEIN1
SLR1-BP1: SLR1-BINDING PROTEIN 1
SLR1-BP2: SLR1-BINDING PROTEIN 2
sPCP: sporophytic pollen coat protein
SRK: S-LOCUS RECEPTOR KINASE
TKPR1/2: TETRAKETIDE ALPHA-PYRONE REDUCTASE 1/2.
