# Supplementary material for: MS1, a direct target of MS188, regulates the expression of key sporophytic pollen coat protein genes in Arabidopsis
Source: J Exp Bot. 2020 May 6;71(16):4877–89. doi: 10.1093/jxb/eraa219 (PMC7410184; doi:10.1093/jxb/eraa219)
Supplement: eraa219_suppl_Supplementary-Data-Files [file eraa219_suppl_supplementary-data-files.pdf]

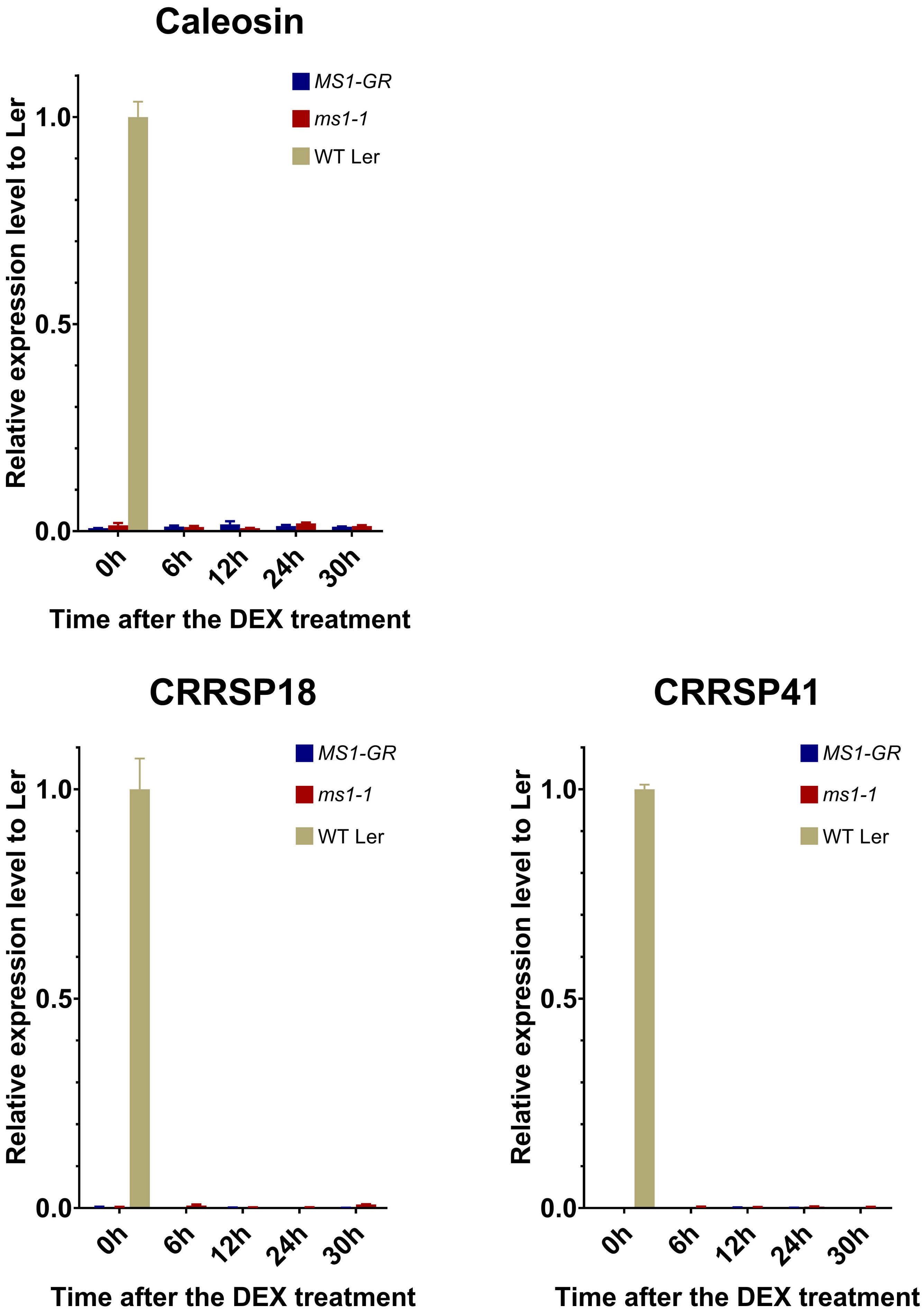

Figure S1. sPCP Genes that showed no significant up-regulation 30h post DEX induction in the MS1-GR line.

A time-course qRT-PCR detection of sPCP Caleosin and CRRSP18, 41 at 6, 12, 24 and 30 hours post DEX treatment in the transgenic line of pMS1:MS1-GR (*ms1-1* background) and the *ms1-1* mutant respectively. The expression data are presented in the same way as in Figure. 4.

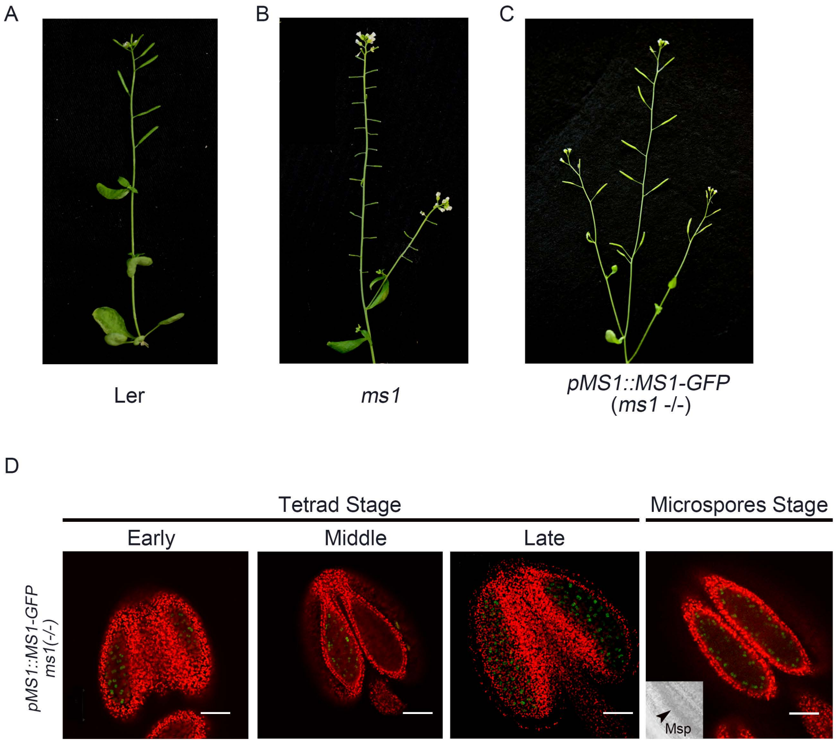

Figure S2. MS1 Expression in *pMS1:MS1-GFP* complementation plants.

(A)-(C) The phenotype of WT (*Ler*-0), *ms1-1*, *pMS1:MS1-GFP* complementation plant respectively and fluorescence confocal images of the MS1-GFP fusion proteins. The green channel shows the GFP expression (530 nm), and the red channel shows the chlorophyll autofluorescence (>560 nm). Bars = 50 μm.

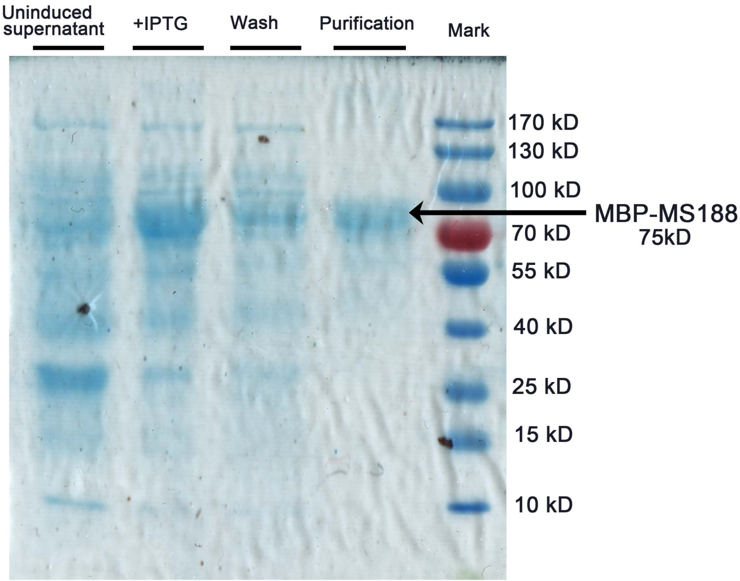

Figure S3. Expression and purification of the MS188 protein.

The MS188 tagged with MBP of 76kD was induced and purified.

Table1 List of primers used in the study and their sequences

Primer	Sequence	Note
CONT F	CCAACCACAACTCCATATTTGTT	For ChIP assays
CONT R	ATAGCTTGACGGCTAGACGCAC	For ChIP assays
Probe^{MS1} F	TTAAGTAAATACCATTGCAATTTGC	For ChIP assays
Probe^{MS1} R	GCATCTATTACATAACACACACGTG	For ChIP assays
Probe-1^{MS1}-Bio F	BIOTIN-TTAAGTAAATACCATTGCAATTTGC	For EMSA assays
Probe-1^{MS1} F	TTAAGTAAATACCATTGCAATTTGC	For EMSA assays
Probe-1^{MS1} R	CGGTTTAAAGAAAGTTTCAAATTAT	For EMSA assays
Probe-2^{MS1}-Bio F	BIOTIN-AATCGACTAAATTGAGTTTGT	For EMSA assays
Probe-2^{MS1} F	AATCGACTAAATTGAGTTTGT	For EMSA assays
Probe-2^{MS1} R	GCATCTATTACATAACACA	For EMSA assays
GRP14 comF	CCGGAATTCAGGAAATAAGTCAAGTTGTTCC	For complementation assays
GRP14 comR	CGCGGATCCCCATGTGGGTGGTGCAGCCGGTT	For complementation assays
GRP17 comF	CGGGGTACCGGATGATCATATGGAGCAACTTG	For complementation assays
GRP17 comR	AACTGCAGCTCCGCCATGTGGCCACCTCCA	For complementation assays
GRP18 comF	CCGGAATTCAGAACAATGGTCAAGTTGTTCCAAC	For complementation assays
GRP18 comR	CGCGGATCCCCATGTGGGTGGTGTACTTCCA	For complementation assays
GRP19 comF	CGCGGATCCCAAGGATGCATGACGATATGGAAA	For complementation assays
GRP19 comR	GCTCTAGAGACGCCGGAACCTGCTGGGTTAGA	For complementation assays
EXL4 comF	CCGGAATTCATTAAAAACTATTTTCACGGC	For complementation assays
EXL4 comR	CGCGGATCCGGCAAGGCCATTCTTGATATCCT	For complementation assays
EXL6 comF	CGCGGATCCTTTCTTACAATGCTATTTTCATC	For complementation assays
EXL6 comR	AACTGCAGGGCAAGGCCTTTCGCTATCTGATA	For complementation assays
MS1 comF	AGAATTCGAAGTGACTGTAAGTACCCAAAG	For complementation assays
MS1 comR	AGTCGACGGGTAAAAAAGAGAGAGGAATAAG	For complementation assays
MWD9 F	TAGGGTCGTGGTTGGTTG	For complementation assays
MWD9 R	CTGGCCTCTCTATCTGATAC	For complementation assays
MS1 JD F	TCGTGGTGGAGTCAGTGGTGGGT	For complementation assays
MS1 JD R	TGGGTGCTCAGGTAGTGGTTGTCG	For complementation assays
MS188-PMAL F	GTCGACATGGGTCGGATTCCATGTTGT	For protein expression
MS188-PMAL R	CTGCAGTCAAACCATATGATTGATGAGATCA	For protein expression
GRP14 QPCR F	CCACGGCATTAACCATAACCACA	Primers of qPCR assays
GRP14 QPCR R	CAAATCTCCTACGCCCGAACCTA	Primers of qPCR assays

GRP16 QPCR F	TTGTCATCTTCAGTCCGATTCTCG	Primers of qPCR assays
GRP16 QPCR R	CCCGGTTGATCTGCTTTGGTTGG	Primers of qPCR assays
GRP17 QPCR F	TAATCTTCGTCGGTTTCGCCTGTG	Primers of qPCR assays
GRP17 QPCR R	TCCTTCGGCTTTACTCCCATCCT	Primers of qPCR assays
GRP18 QPCR F	CCACGGCATTAACCATAACAACAC	Primers of qPCR assays
GRP18 QPCR R	GCAGTCGGCTTAGCTCCCATCCT	Primers of qPCR assays
GRP19 QPCR F	AGTGGTCGCATGTATCATAAGCA	Primers of qPCR assays
GRP19 QPCR R	GTAGGTCCCTCCCTGTACGTTTC	Primers of qPCR assays
EXL4 QPCR F	CAGTACAACGGAAAATTGAGGAG	Primers of qPCR assays
EXL4 QPCR R	CGTAATCATACAACAACACCCAT	Primers of qPCR assays
EXL6 QPCR F	CGGTACTCTTATGGATCTTGTCA	Primers of qPCR assays
EXL6 QPCR R	ATATTACTTCGTAGGCTTTCTGG	Primers of qPCR assays
CRRSP18 QPCR F	CATCACCTACCAGTGCCGTATCGA	Primers of qPCR assays
CRRSP18 QPCR R	GATCTGCCCAGAAGTATCTAACG	Primers of qPCR assays
CRRSP41 QPCR F	CCCTTGCATTAGCACCGCCGTCTC	Primers of qPCR assays
CRRSP41 QPCR R	TCACTCATGTTGTTTGGGTTGGA	Primers of qPCR assays
Caleosin QPCR F	AAATTCGAGGAAATATTCAACAAG	Primers of qPCR assays
Caleosin QPCR R	GGAAAAGGCTACCATCGTAGATG	Primers of qPCR assays
MS1 QPCR F	TGTCCTTCACGGCTTCTTTC	Primers of qPCR assays
MS1 QPCR R	CGCGTCATTCAACCCTATTT	Primers of qPCR assays
β-tublulin QPCR F	GACACTACACTGAAGGTGCTGAG	Primers of qPCR assays
β-tublulin QPCR R	AAGCTGATGAACAGAGAGAGTTG	Primers of qPCR assays
MS1 RT-PCR F	ACGGATTTGGTCACTTGCTC	Primers of RT-PCR assays
MS1 RT-PCR R	CTCGGTTAAGGCTGGTTAGG	Primers of RT-PCR assays
β-tubulin RT-PCR F	GGACACTACACTGAAGGTGCTGAG	Primers of RT-PCR assays
β-tubulin RT-PCR R	CAAGCTGATGAACAGAGAGAGTTG	Primers of RT-PCR assays
GRP14 RT-PCR F	GTGGTTATTGCTGCCGTGGTCTC	Primers of RT-PCR assays
GRP14 RT-PCR R	TTTCGCCTCCTCCTAATCCTCCA	Primers of RT-PCR assays
GRP16 RT-PCR F	TTATCGCTGCCGTAGCATCTGTA	Primers of RT-PCR assays
GRP16 RT-PCR R	TCCTCCCGGTTTATCTCCTCCTC	Primers of RT-PCR assays
GRP17 RT-PCR F	TAATCTTCGTCGGTTTCGCCTGTG	Primers of RT-PCR assays
GRP17 RT-PCR R	CCTTCGGACTTACCGCCTTTACC	Primers of RT-PCR assays
GRP18 RT-PCR F	GCTACCGTGGTCTCCATAGTCTTCTT	Primers of RT-PCR assays
GRP18 RT-PCR R	CCTGGTTTATTTCCGAATGTTCCA	Primers of RT-PCR assays
GRP19 RT-PCR F	GTTTGAGATTATTCAGGCGGTCTTC	Primers of RT-PCR assays

GRP19 RT-PCR R	GGTAGGTCCCTCCCTGTACGTTTC	Primers of RT-PCR assays
EXL4 RT-PCR F	AACGCCAAAGGATCAAGTAAACG	Primers of RT-PCR assays
EXL4 RT-PCR R	GATGACGAACCCACCAAGGAAGA	Primers of RT-PCR assays
EXL6 RT-PCR F	TTTTATCGGCAGGTGACCAAGTA	Primers of RT-PCR assays
EXL6 RT-PCR R	GAAGTTACACCATCCGAATACCC	Primers of RT-PCR assays
CRRSP18 RT-PCR F	GTTCTAGTCCCTATTGTGGTCGTG	Primers of RT-PCR assays
CRRSP18 RT-PCR R	AGTTGTCGTCGTAATTGATCTGC	Primers of RT-PCR assays
CRRSP41 RT-PCR F	ATAGCCATACAACTCCTCCTTACA	Primers of RT-PCR assays
CRRSP41 RT-PCR R	TTGGGTTGGACAAATAGAAATCG	Primers of RT-PCR assays
Caleosin RT-PCR F	ATTTGTGGAATCAAAATTCGAGG	Primers of RT-PCR assays
Caleosin RT-PCR R	CTGACGCTCTTTTCCGATAACAA	Primers of RT-PCR assays
Caleosin QPCR-DEX F	AAGGGATTCTCGCCGTTGTTTC	For DEX induction assays
Caleosin QPCR-DEX R	TCCACAAATCTTCCGTCATCGTC	For DEX induction assays
EXL4 QPCR-DEX F	GCGGTAATGGGAGTGATACCGTTG	For DEX induction assays
EXL4 QPCR-DEX R	ACGTGATGACGAACCCACCAAG	For DEX induction assays
EXL5 QPCR-DEX F	GGCAATAGTACCATGCTCCAACC	For DEX induction assays
EXL5 QPCR-DEX R	TGTAGGCTTTCTCGGAGGGATGAG	For DEX induction assays
EXL6 QPCR-DEX F	TGTCGCTCCCGCTCTTCTTC	For DEX induction assays
EXL6 QPCR-DEX R	TCCGAATACCCCACCGAATG	For DEX induction assays
GRP14 QPCR-DEX F	CGACGTAGGTTCGGGCGTAG	For DEX induction assays
GRP14 QPCR-DEX R	AGCCCCTGGTCCGAACATTT	For DEX induction assays
GRP16 QPCR-DEX F	ATCGCTGCCGTAGCATCTGTAG	For DEX induction assays
GRP16 QPCR-DEX R	TCCCACAATTGAGGCACCGAAG	For DEX induction assays
GRP17 QPCR-DEX F	TGTCTTGGCGACAGGATTCACG	For DEX induction assays
GRP17 QPCR-DEX R	CTCCCATCCTACGTTTAACAAGCC	For DEX induction assays
GRP18 QPCR-DEX F	CGTTGCTCTCATCAGACGTAGG	For DEX induction assays
GRP18 QPCR-DEX R	TTGGGCTGACGAAGTTCCTTCC	For DEX induction assays
GRP19 QPCR-DEX F	AGCCACTACATTGCTAGCTTCAGG	For DEX induction assays
GRP19 QPCR-DEX R	AGGTCCCTCCCTGTACGTTTCTTG	For DEX induction assays
GRP20 QPCR-DEX F	TGCTGGCCGGTATATCTCTAACC	For DEX induction assays
GRP20 QPCR-DEX R	ATAGCGGCATGGACGCGAATAG	For DEX induction assays
GRP-Oleosin QPCR-DEX F	AGGAACTTGGACAGCAAATTGGG	For DEX induction assays
GRP-Oleosin QPCR-DEX R	ACCGCCAGACTGATCTCCAAAC	For DEX induction assays
CRRSP18 QPCR-DEX F	CGAAGAACCTGATCGGCAATTC	For DEX induction assays
CRRSP18 QPCR-DEX R	AGCCGAACATTGCACCATTC	For DEX induction assays

CRRSP41 QPCR-DEX F	TCCACAGTGTTGTGACGGTAAACG	For DEX induction assays
CRRSP41 QPCR-DEX R	GGAGCTCTCATTGTACTGTTGCTG	For DEX induction assays
ATA27/BGLU20 QPCR-DEX F	ATTCTCTCGTGGACTGGGAACC	For DEX induction assays
ATA27/BGLU20 QPCR-DEX R	AGAGGCTCCTCAAACCCTTTGC	For DEX induction assays
MYB99 QPCR-DEX F	CATGCTCGCCTTGGCAATAGATG	For DEX induction assays
MYB99 QPCR-DEX R	GTCTGTTCTTCCTGGTAACTCCAC	For DEX induction assays
ProMS188 F	CGAGCTCAAGTTGTGTTTTTTCCCAAGTCA	For rescue trangenic assay
ProMS188 R	TCCCCCCGGGTTCTTCTTTCTTTCTTTCTAGTTTTT	For rescue trangenic assay
MS1 Genomic F	TCCCCCCGGGATGGCGAATCTGATTCGAA	For rescue trangenic assay
MS1 Genomic R	ACGCGTCGACTTAGGGTAAAAAAGAGAGAGGAA	For rescue trangenic assay
Pro MS1 LUCF	AACTGCAGCCGCTACTGGCAATTTCAAT	Transient activating assays
Pro MS1 LUCR	CGGGATCCCGAATCAGAAATTTGGTTTGATC	Transient activating assays
MS188 Overexpress F	GCTCTAGAATGGGTCGGATTCCATGTT	Transient activating assays
MS188 Overexpress R	ACGCGTCGACAACCATATGATTGATGAGATCATCA	Transient activating assays
